# Impact of heart rate in atrial fibrillation versus sinus rhythm on mortality in octogenarian patients with acute coronary syndrome

**DOI:** 10.11604/pamj.2017.28.89.11170

**Published:** 2017-09-29

**Authors:** Shijun Li, Salim Barywani, Michael Fu

**Affiliations:** 1 Department of Geriatric Cardiology, Chinese PLA General Hospital, Beijing, China; 2 Section of Cardiology, Department of Medicine, Sahlgrenska University Hospital/Östra Hospital, Gothenburg, Sweden

**Keywords:** Heart rate, atrial fibrillation, mortality, octogenarian, acute coronary syndrome

## Abstract

**Introduction:**

Association of heart rate (HR) with mortality in patients with acute coronary syndrome (ACS) and aged ≥ 80 years are underrepresented in clinical trials. We therefore aimed to investigate the association of HR in atrial fibrillation (AF) versus sinus rhythm (SR) with all-cause mortality in octogenarian patients with ACS.

**Methods:**

A total of 336 patients with ACS patients and aged ≥ 80 years were enrolled into the current study. The end point of interest was death from any cause. Association of HR in AF versus SR with mortality was analyzed by Kaplan-Meier curve following log-rank test and multivariable Cox regression analysis.

**Results:**

In total, 63 (87.5%) of patients with AF were dead and 147 (59.8%) of patients with SR were dead during the follow-up period. The best cut-off was 80 bpm, with a sensitivity of 62% and specificity of 66%. HR ≤ 80 bpm in SR but not in AF was associated with better outcome as compared with HR > 80 bpm (Chi-Square = 26.55, Log rank P < 0.001). In SR subgroup, the hazard ratios of HR ≤ 80 bpm were 0.51(95% CI 0.37-0.70, P < 0.001) adjusted for age, 0.46 (95%CI 0.33-0.63, P < 0.001) adjusted for gender, 0.62 (95%CI 0.42- 0.93, P = 0.020) adjusted for multivariables respectively. In AF subgroup, the hazard ratios of HR ≤ 80 bpm were 0.83(95% CI 0.49-1.38, P = 0.464) adjusted for age, 0.96 (95%CI 0.59-1.58, P = 0.882) adjusted for gender, 0.72(95% CI 0.41-1.26, P = 0.249) adjusted for multivariables respectively.

**Conclusion:**

The current study demonstrates that heart rate is an independent prognostic predictor for all-cause mortality, and HR ≤ 80 bpm is associated with improved outcome in SR but not in AF in octogenarian patients with ACS.

## Introduction

Advanced age is associated with greater prevalence and severity of coronary artery disease and higher risk of ischemic complications and mortality, and 30% of deaths related to myocardial infarction occur in patients aged > 85 years [1]. Moreover, most deaths in patients aged ≥ 75 years are of ischemic origin [2]. Despite of the increased complexity of management in very elderly patients with acute coronary syndrome (ACS), data on those aged ≥ 80 years are limited, since these patients are often excluded from clinical trials [1, 3]. Current guidelines for this population are thus based on extrapolation of data for younger patients, which may not be applicable to the advanced ages. How to better management for these octogenarian patients with ACS is becoming more and more pressing. Heart rate control is currently being considered to be an effective management for patients with ACS. An increasing resting HR might be a marker or even a risk factor for cardiovascular morbidity and mortality [4–7]. Admission heart rate (HR) values could independently predict mortality in patients with ST-elevation acute myocardial infarction (STEMI) [8–11]. In the context of non- STEMI, the relationship between presenting HR and in-hospital cardiovascular outcomes has a ‘J-shaped’ curve (higher event rates at very low and high HRs) [12]. However, impact of HR on mortality isn’t adequately studied in the overall ACS setting. Limited data showed that low (< 60 bpm) or high HR (≥ 70bpm, ≥ 80bpm or ≥ 90bpm) is a marker of high risk that needs more attention and management, and higher HR is highly predictive of higher short- and long-term mortality in patients with ACS [13–16]. However these studies were conducted in ACS patients aged < 80 years, and their results weren’t further analyse in sinus rhythm (SR) or a trial fibrillation (AF) subgroup and the cut off value of HR was mostly pre-defined before the study was conducted. The impact of heart rate in AF versus SR on mortality in octogenarian ACS patients isn’t adequately studied. We therefore aimed to evaluate the association of HR in AF versus SR with all-cause mortality in octogenarian patients in the overall ACS setting.

## Methods

### Study design

The in-hospital patients (n = 353) aged ≥ 80 years with ACS were enrolled from January 2003 to December 2007. Acute myocardial infarction was defined by at least 2 of the following features [17]: 1) electrocardiographic changes indicative of ischemia (ST-segment elevation or depression); 2) compatible clinical symptoms; and 3) specific diagnostic biomarker elevations (troponin I > 0.4 ng/ml and serum creatine kinase-MB isoenzyme (CK-MB) > 8.8 ng/ml). Unstable angina was defined by the occurrence of 1 or more angina episodes, at rest, within the preceding 48 h, corresponding to class III of the Braunwald classification [18]. The exclusion criteria were: stable angina pectoris and inaccessible medical data, and a missing discharge status or leaving against medical advice during the follow-up period. All hospitalized patients received the optimal therapy according to their clinical status. We didn’t enrolled the patients with pacing rhythm or the other rhythms rather than SR or AF, and excluded 17 (4.8%) due to a missing discharge status. The final study population included 336 (95.2%) in this current study.

### Clinical assessments

We collected data on clinical characteristics, past medical history, comorbidities, heart rate and blood pressure indicators, therapies, interventions, and outcomes. All hospitalized patients received the optimal therapy according to their clinical status. The HR was obtained from the first measurement record by using electrocardiogram (ECG) after presentation to the emergency department or the hospital ward. The presence of AF at the time of presentation was based on ECG data and documentation in the medical records.

### Outcomes

The endpoint of interest was death from any cause. Survival status was ascertained on 1 December, 2012 by cross-referencing the Cause of Death Register maintained by the National Board of Health and Welfare (a Swedish government agency).

### Ethics approval

This study was approved by the Human Subjects Review Committee at the Sahlgrenska University Hospital/Östra Hospital in Gothenburg, Sweden.

### Statistical analyses

Categorical variables are expressed as percentages, and continuous variables are expressed as the mean ± SD. All continuous variables were first tested for normality and homogeneity of variance. Patients were grouped by heart rate, and differences in baseline characteristics were tested using Pearson chi-square tests for categorical variables and a one-way analysis of variance (ANOVA) for continuous variables with normality and Kruskal-Wallis H rank-sum tests for continuous variables with non-normality.

Receiver operator characteristics (ROC) curve analysis was performed to study the predictive value of admission HR on the considered mortality endpoints and to select the best cut off (maximizing the sum of (sensitivity and specificity -1)). The area under the curve (AUC) was used as a measure of the predictive accuracy of HR.

Missing data were handled by multiple imputations with a run with automatic settings, and the method actually chosen by automatic method selection was Fully Conditional Specification. The variables were listed in the imputation sequence order. Scale variables were modelled with a linear regression, categorical variables with a logistic regression, and each model used all other variables as main effects. Multiple imputations were carried out with 5 imputations and 10 iterations per imputation. Kaplan-Meier methods were used to estimate mortality and log-rank tests to assess differences in mortality in HR subgroups. Cox proportional hazards models were used to examine univariate and multivariable associations of HR strata with mortality rate. Differences were considered statistically significant at P < 0.05. Statistical analyses were performed with IBM SPSS Statistic 21.0 (IBM Corporation, Armonk, NY, USA).

The variables which were statistically significance (*P* < 0.05) by univariate Cox regression and interacted items with HR obtained from multivariate Cox regression (*P* < 0.05) were included in final multivariate Cox regression model. Due to some missing variables existing, a consistency analysis was performed excluding patients with missing variables with multivariate Cox regressions.

## Results

### Baseline characteristics

**Figure 1:**
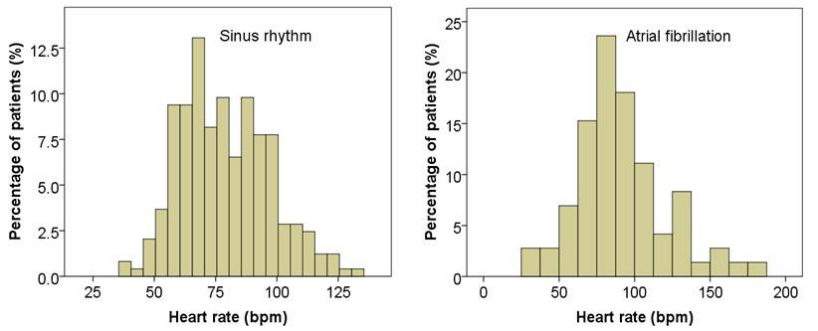
Heart rate distribution in sinus rhythm and atrial fibrillation

We evaluated 336 patients with ACS during median 41.5-month (interquartile range (IQR): 5 to 64 months) follow-up period. Of these, 173 (51.5%) were men and 163 (48.5%) were women, and 177 (52.7%) were admitted for STEMI, 112 (33.4%) for NSTEMI and 47 (14.0%) for unstable angina. The demographic and baseline characteristics in HR strata were summarized in Table 1. The distributions of sinus rhythm (SR) and AF among these HR groups are shown in Figure 1. In total, 72 (21.4%) were with AF and 246(73.2%) were with SR. There was no significant difference in the incidence rate of STEMI among different HR strata. The number of patients with AF was gradually increases, whereas ejection fraction and PCI operation were gradually decreased with heart rate increment (Table 1).

**Table 1:** Baseline characteristics

	Missing rate (%)	HR ≤ 70 bpm (n=118 )	HR70-90 bpm (n=110 )	HR > 90 bpm (n=108 )	P value
**Characteristics**					
Age, yrs	0	85.28(3.99)	85.41(3.94)	85.73(4.46)	0.860
Male gender	0	68(57.6)	57(51.8)	48(44.4)	0.140
BMI, kg/m2	24.7	24.74(3.54)	24.28(3.85)	24.37(4.45)	0.410
Smoker	8.0	4(3.6)	10(10.0)	6(6.1)	0.167
Systolic BP, mmHg	3.6	147.26(27.71)	146.94(25.57)	148.66(27.79)	0.888
Diastolic BP, mmHg	3.9	80.00(15.04)	84.13(14.78)	87.57(15.99)	0.002
Ejection fraction, %	34.5	49.21(9.70)	46.52(10.53)	44.55(11.86)	0.030
**Medical history**					
STEMI	0	61(51.4)	58(52.5)	58(53.4)	0.794
Hypertension	0.3	76(64.4)	68(62.4)	79(73.1)	0.201
Hypercholesterolemia	0.3	12(10.2)	12(11.0)	17(15.7)	0.395
Diabetes	4.5	13(11.8)	23(21.9)	28(26.4)	0.023
Atrial fibrillation	5.4	19(16.7)	21(20.0)	32(32.3)	0.018
Prior renal failure	5.7	9(8.1)	10(9.7)	10(9.7)	0.895
Prior heart failure	7.1	25(23.4)	31(30.1)	27(26.5)	0.543
Prior anemia	4.8	12(10.8)	8(7.7)	18(17.1)	0.098
Prior stroke	3.3	16(14.0)	18(17.1)	26(24.5)	0.123
**Laboratory tests**					
eGFR, ml/min	20.2	48.54(17.52)	48.79(21.90)	47.20(19.45)	0.811
Haemoglubin	13.1	133.37(16.01)	130.00(17.75)	127.24(18.04)	0.047
Sodium	12.8	139.09(4.48)	138.88(3.51)	138.00(7.39)	0.607
Potassium	12.2	4.19(0.53)	4.26(0.54)	4.15(0.51)	0.229
**Medications**					
Antiplatelets	0.1	105(97.2)	88(89.8)	87(95.6)	0.058
Warfarin	11.0	7(6.5)	7(7.1)	7(7.6)	0.953
Beta blocker	11.9	90(83.3)	91(93.8)	84(92.3)	0.029
ACEIs	11.9	37(34.9)	51(51.5)	44(48.4)	0.039
ARBs	11.3	9(8.4)	8(8.1)	9(9.8)	0.908
CCBs	11.3	29(26.9)	26(26.3)	19(20.9)	0.575
Statins	11.3	67(62.0)	58(58.6)	43(47.3)	0.096
Loop diuretics	11.6	44(41.1)	51(51.5)	60(65.9)	0.002
Spironolactone	12.8	8(7.6)	9(9.3)	9(9.9)	0.844
Digoxin	3.9	10(8.8)	9(8.6)	14(13.3)	0.438
Antidepressants	0.02	17(14.8)	23(21.3)	15(14.0)	0.286

Notes: Data are expressed in either mean (SD) or n (%). Notes: HR, heart rate, BP, blood pressure; BMI, body mass index; eGFR, estimated glomerular filtration rate; STEMI, ST elevation myocardial infarction; ACEIs, angiotensin converting enzyme inhibitors; ARBs, angiotensin II receptor blockers; CCBs, calcium channel blockers.

### Outcomes

**Figure 2:**
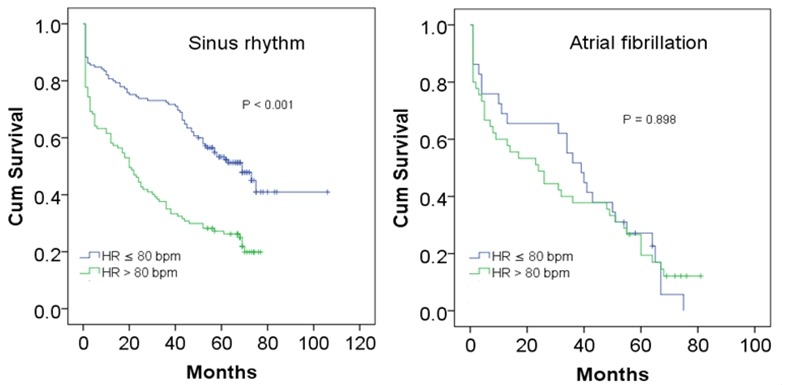
Kaplan-Meier curves for mortality in patients with ACS and AF or SR according to heart rate strata

In total, 63 (87.5%) of patients with AF were dead and 147 (59.8%) of patients with SR were dead during the follow-up period (Table 1). Moreover, the mortality rate was obviously increased, and the survival rate was significantly decreased when HR was greater than 80 bpm in overall cohort. By ROC curve analysis, we obtained an AUC of 0.63 (95% CI: 0.56-0.69, P < 0.001) for HR to predict all-cause mortality, and the best cut-off was 80 bpm, with a sensitivity of 62% and specificity of 66%. 4HR ≤ 80 bpm in SR exhibited better outcome as compared with HR > 80 bpm (Chi-Square = 26.55, Log rank P < 0.001), whereas in AF, no significant difference in mortality was found among different HR strata (Chi-Square = 0.02, Log rank *P* = 0.898) by Kaplan-Meier analyses (Figure 2). Multivariate Cox regression showed that HR was an independent predictor for mortality in overall cohort and subgroup with SR (All P < 0.001). In overall cohort, the hazard ratios of HR ≤ 80 bpm were 0.55 (95% CI 0.42-0.72, P < 0.001) adjusted for age, 0.52 (95% CI 0.40-0.68, P < 0.001) adjusted for gender, 0.66(95% CI 0.47-0.90, P = 0.009) adjusted for multivariable respectively. In SR subgroup, the hazard ratios of HR ≥ 80 bpm were 0.51(95% CI 0.37-0.70, *P* < 0.001) adjusted for age, 0.46(95%CI 0.33-0.63, *P* < 0.001) adjusted for gender, 0.62(95%CI 0.42-0.93, P = 0.020) adjusted for multivariable respectively. In AF subgroup, the hazard ratios of HR ≥ 80 bpm were 0.83(95% CI 0.49-1.38, P = 0.464) adjusted for age, 0.96(95%CI 0.59-1.58, P = 0.882) adjusted for gender, 0.72(95% CI 0.41-1.26, *P* = 0.249) adjusted for multivariable respectively (Table 2).

**Table 2:** Cox regression analyses for impact of heart rate on mortality with imputation

Heart rate, ≤ 80 bpm vs. > 80 bpm			
Model	Hazard ratio	95% CI	*P*-value
*Overall cohort*			
Univariable	0.52	0.40-0.68	< 0.001
Adjusted for			
Age	0.55	0.42-0.72	< 0.001
Gender	0.52	0.40-0.68	< 0.001
Adjusted for multivariables^+^	0.66	0.47-0.90	0.009
*SR subgroup*			
Univariable	0.46	0.34-0.63	< 0.001
Adjusted for			
Age	0.51	0.37-0.70	< 0.001
Gender	0.46	0.33-0.63	< 0.001
Adjusted for multivariables ^†^	0.62	0.42-0.93	0.020
*AF subgroup*			
Univariable	0.96	0.59-1.58	0.881
Adjusted for			
Age	0.83	0.49-1.38	0.464
Gender	0.96	0.59-1.58	0.882
Adjusted for multivariables ^#^	0.72	0.41-1.26	0.249

Notes: Adjustment for the variables with statistical significance by univariable Cox regression and items interacted with heart rate:

+ including age, smoker, systolic BP, diastolic BP, LVEF, diabetes, prior renal failure, prior heart failure, prior stroke, prior CABG, eGFR, haemoglubin, potassium, beta blocker, statins, loop diuretics, antidepressants and PCI, and interacted item of heart rate, heart rate×atrial fibrillation;

† including age, smoker, systolic BP, diastolic BP, LVEF, diabetes, prior renal failure, prior heart failure, prior stroke, prior CABG, eGFR, haemoglubin, beta blocker, statins, loop diuretics and PCI, and interacted items of heart rate, heart rate×diabetes;

# including age, prior heart failure, eGFR and loop diuretics in multivariable Cox regression.

In sensitivity analyses without missing variables, heart rate ≤ 80 bpm was still related to better outcome in overall patients or in SR subgroup but not in AF subgroup by using multivariable Cox regression analyses (Table 3).

**Table 3:** sensitivity analyses for impact of heart rate on mortality with multivariable Cox regressions in patients without missing variables

Heart rate, ≤ 80 bpm vs. > 80 bpm			
Model	Hazard ratio	95% CI	*P*-value
*Overall cohort*			
Univariable	0.45	0.28-0.71	0.001
**Adjusted for**			
Age	0.40	0.25-0.6	< 0.001
Gender	0.45	0.29-0.71	0.001
Adjusted for multivariables^+^	0.38	0.18-0.77	0.007
***SR subgroup***			
Univariable	0.34	0.19-0.60	< 0.001
**Adjusted for**			
Age	0.31	0.17-0.55	< 0.001
Gender	0.35	0.20-0.62	< 0.001
Adjusted for multivariables ^†^	0.31	0.12-0.84	0.021
***AF subgroup***			
Univariable	1.23	0.55-2.76	0.610
**Adjusted for**			
Age	1.32	0.53-3.25	0.552
Gender	1.22	0.55-2.74	0.628
Adjusted for multivariables ^#^	2.38	0.80-7.07	0.119

Notes: Adjustment for the variables with statistical significance by univariable Cox regression and items interacted with heart rate:

+ including age, smoker, systolic BP, diastolic BP, LVEF, diabetes, prior renal failure, prior heart failure, prior stroke, prior CABG, eGFR, haemoglubin, potassium, beta blocker, statins, loop diuretics, antidepressants and PCI, and interacted item of heart rate, heart rate×atrial fibrillation;

† including age, smoker, systolic BP, diastolic BP, LVEF, diabetes, prior renal failure, prior heart failure, prior stroke, prior CABG, eGFR, haemoglubin, beta blocker, statins, loop diuretics and PCI, and interacted items of heart rate, heart rate×diabetes;

# including age, prior heart failure, eGFR and loop diuretics in multivariable Cox regression.

## Discussion

The current study reported the association of HR in AF versus SR with mortality in octogenarian patients with ACS. The principal findings are that admission heart rate is an independent predictor for all-cause mortality, and HR ≥ 80 bpm is associated with improved all-cause mortality in patients in SR but not in AF. Advanced age is an important risk factor for acute coronary syndrome (ACS). The very old patients with ACS often carried more co-morbidities such as AF, less early aggressive treatment and worse outcomes. Some data showed that AF occurs in about 5–20% of patients with ACS and both AF and ACS are common in patients of advanced age, which results in further poor prognosis [19–21]. In our study, 21.4% of patients with ACS and aged ≥ 80 years were with AF, and 87.5% of patients with AF were dead during the median 41.5-month follow-up period. Previously, every effort was made to restore and maintain SR for patients with AF, but it turned out to be difficult to keep SR in the long-term [22–24]. Recent studies showed that rate control was not inferior to rhythm control with regard to cardiovascular morbidity and mortality [25–27], rate control has therefore been adopted as the front-line therapy in many patients with AF.

The Framingham Heart study and the French IPC study showed that resting HR was associated with cardiovascular mortality in the general population [4, 28]. Evidence showed that admission heart rate (HR) values could independently predict mortality in patients with STEM or non-STEMI [8–12]. In the context of the overall ACS, a high HR had been shown to be an independent predictor of short-term (30 days) and medium-term (one year) prognoses in a population with ACS [13–16]. In ACS prognostic models, such as the PURSUIT and GRACE risk models, there was a linear association between heart rate and cardiovascular outcomes [29, 30]. However, these previous studies were conducted in ACS patients aged < 80 years, and their results weren’t further analysed in SR or AF subgroup. Evidence showed that medications that are beneficial in lowering the heart rate and improving outcomes for patients in SR have not been shown to have similar value in patients with AF [31]. Similarly, findings from the CHARM programme suggested that high heart rates were not predictive of chronic heart failure outcomes in patients in AF compared to those in SR [32]. However, the impact of HR in AF versus SR on mortality in octogenarian patients isn’t still adequately studied in the overall ACS setting. The current study showed that the mortality rate was significantly increased when HR was less than 80 bpm, and the best cut-off was 80 bpm with a sensitivity of 62% and specificity of 66%. Furthermore, HR was an independent predictor for mortality, and HR ≤ 80 bpm was associated with improving outcome in SR but not in AF subgroup. The sensitivity analyses further confirm the results.

Our study has some limitations. First, there are some missing values in the dataset, and we performed multiple imputations to reduce bias and retain statistical power. Second, to avoid potential confounding due to excessive imputation, we performed sensitivity analyses in patients without missing value. Third, the time point which AF was defined was at enrolment, so we were unable to differentiate new-onset AF and chronic atrial fibrillation and we were unable to separate paroxysmal, persistent and permanent AF in our database. At last, the present data provides no insight into the impact of resting heart rate in the healthy octogenarian population and haven’t provided with regard to cause specific mortality from cardiovascular disease or non-cardiovascular disease.

## Conclusion

The current study demonstrates that admission heart rate is an independent predictor for all-cause mortality and HR = 80 bpm is associated with improved outcome in patients in SR but not in AF in octogenarian patients with ACS.

### What is known about this topic

- The increasing burden of elderly patients on health care resources stresses the need for research focused specifically on this part of the population. Nevertheless, elderly patients are underrepresented in clinical trials;
- The emerging data supported that heart rate might be a marker or even a risk factor for cardiovascular morbidity and mortality. However the independent contribution of admission HR to long-term all-cause mortality and whether the relationship was modified by AF remain unknown in the octogenarian ACS.

### What this study adds

- This study provides important insight into the relationship between admission HR and long-term all-cause mortality in octogenarian ACS patients, and demonstrated that HR ? 80 bpm can independently predict the long-term all-cause mortality in octogenarian ACS patients;
- The presence of AF can alter the relationship between HR ≤ 80 bpm and the long-term mortality, and HR ≤ 80 bpm have the prognostic impact of on the long-term mortality only in those with SR but not in those with AF.

## Competing interests

The authors declare no competing interests.
